# Controlled growth of hexagonal gold nanostructures during thermally induced self-assembling on Ge(001) surface

**DOI:** 10.1038/srep42420

**Published:** 2017-02-14

**Authors:** B. R. Jany, N. Gauquelin, T. Willhammar, M. Nikiel, K. H. W. van den Bos, A. Janas, K. Szajna, J. Verbeeck, S. Van Aert, G. Van Tendeloo, F. Krok

**Affiliations:** 1 Marian Smoluchowski Institute of Physics Jagiellonian University, Lojasiewicza 11, PL-30348 Krakow, Poland; 2 EMAT University of Antwerp, Groenenborgerlaan 171, BE-2020 Antwerp, Belgium

## Abstract

Nano-sized gold has become an important material in various fields of science and technology, where control over the size and crystallography is desired to tailor the functionality. Gold crystallizes in the face-centered cubic (*fcc*) phase, and its hexagonal closed packed (*hcp*) structure is a very unusual and rare phase. Stable Au *hcp* phase has been reported to form in nanoparticles at the tips of some Ge nanowires. It has also recently been synthesized in the form of thin graphene-supported sheets which are unstable under electron beam irradiation. Here, we show that stable *hcp* Au 3D nanostructures with well-defined crystallographic orientation and size can be systematically created in a process of thermally induced self-assembly of thin Au layer on Ge(001) monocrystal. The Au *hcp* crystallite is present in each Au nanostructure and has been characterized by different electron microscopy techniques. We report that a careful heat treatment above the eutectic melting temperature and a controlled cooling is required to form the *hcp* phase of Au on a Ge single crystal. This new method gives scientific prospects to obtain stable Au *hcp* phase for future applications in a rather simple manner as well as redefine the phase diagram of Gold with Germanium.

Gold is a noble material and has been extensively used in many areas of science and technology such as nanoelectronics^1^,^2^, catalysis^3^, nanophotonics^4^ and medicine^5^, for its unique properties^6^,^7^ (non-reactive, highly conductive, biocompatible) over the last decades. The hexagonal closed packed (*hcp*) structure of Au is a very rare form of gold as it naturally occurs in the cubic face centered (*fcc*) phase. Single-phase crystals of this unusual form of gold were solely obtained as graphene-supported nanometer-thick Au_hcp_ sheets^8^ or as nanoribbons^9^ by wet chemistry synthesis, but so far little is known about its properties. It is important to remark that the Au_hcp_ nanosheeets were reported by the authors to be unstable under electron irradiation reverting their structure to the conventional Au_fcc_ phase^8^,^9^. Recently, Marshall *et al*.^11^,^12^ reported on synthesis of stable Au *hcp* nanoparticles on top of some germanium nanorods via quenching of Au/Ge liquid. Only ~30% of the resulted nanoparticles were made of Au *hcp* phase. Since this form of Au is so rare and very difficult to obtain, its properties were never studied. Nonetheless it was theoretically explored recently by Wang *et al*.^13^, who has shown the possibility of *hcp* to fcc phase transition by first principle calculations. The elastic constants for Au *hcp* were also calculated, confirming the possibility of fabricating pure and stable *hcp* Au under ambient conditions. The method we propose here gives the possibility to synthesize stable 3D Au_hcp_ nanostructures with tunable size via thermally-induced Au self-assembly on a Ge(001) surface. In our work, the Au_hcp_ crystallite is present in each formed nanostructure as evidenced by electron microscopy i.e. Electron-Back-Scattered Diffraction (EBSD) and high-angle annular dark-field scanning transmission electron microscopy (HAADF-STEM). This gives unique scientific prospects to obtain in a simple way a stable Au_hcp_ phase for future applications and studies in microelectronics or catalysis. The Au/Ge(001) surface itself is also very interesting for the applications of future mono-molecular devices since it may exhibit 1D and 2D conductivity channels in the form of atomic chains^1^ and a subsurface layer^2^. The deposited Au thin films show a preferential growth in the <110> direction when deposited on (001) germanium single crystal surfaces^14^ and annealed at temperatures below the eutectic point of the Au/Ge system of ~634 K^15^. As the annealing temperature of the sample reaches ~634 K, part of the germanium substrate melts and forms a Au-Ge eutectic liquid, which then recrystallizes during cooling of the sample to room temperature (RT)^15^. Here, the structure and composition of nanostructures formed after RT deposition of 6 mono layers (ML) of Au on (2 × 1) reconstructed Ge(001) in UHV^16^ and subsequent post-annealing at different temperatures (below and above the eutectic melting temperature of Au/Ge (634 K)–*T*_*e*_) and cooling rates were investigated using a combination of electron microscopy techniques. The structure and formation of Au nanoparticles on a single crystal have garnered a renewed interest in nanotechnology^6^ as these are the key catalysts in the growth of Ge, Si and III/V nanowires with tremendous applications for a new generation of nanoscale electronics and optoelectronics devices such as solar cells and battery electrodes^17^. The formed Au *hcp* 3D nanostructures could be also used as a growth template for hexagonal semiconductors^18^.

In the present study, Au islands of rectangular shape with edges oriented along [110] and [1–10] directions of the Ge(001) substrate (called Ge <110> in the rest of the text) are created for a post-annealing temperature of 573 K (bellow *T*_*e*_), as seen in Fig. 1a. When the post-annealing temperature exceeds *T*_*e*_, the islands reshape into an asymmetric polyhedral (see Supplementary Fig. S1), indicating the formation of a AuGe eutectic liquid during the high-temperature stage of the treatment^19^. The longest edge of the island is aligned along the Ge <110> direction (as observed in Fig. 1b). EBSD measurements are performed to determine the crystallographic orientation and phase of each island. It is important to realize that the EBSD technique probes the subsurface region down to a depth of a few nanometers for gold. In Fig. 1(c,d), the resulting inverse pole figures (IPF) in the *Z* (out of plane) sample direction of the EBSD measurements for the sample post-annealed bellow (Fig. 1c) and above (Fig. 1d) *T*_*e*_ are shown, respectively. For a low temperature, the Au crystallites were indexed unequivocally as belonging to the Au_fcc_ structure, while for the sample annealed at a higher temperature, the Au crystallites could not be indexed in the cubic system, and the Au_hcp_ phase has to be considered. Common orientations, independent of the annealing temperatures, of the Au crystallites are observed in the *X, Y* and *Z* sample directions, as depicted in Fig. 1e. For the case of temperatures lower than *T*_*e*_, the obtained EBSD results can be described as being constituted by two equivalent configurations of Au crystallites rotated by 90° in-plane, due to the substrate surface symmetry, (for details see Supplementary Figs S2 and S3, experimental and simulated pole figures and inverse pole figures) with epitaxial relation (101)Au_fcc_//(001)Ge and [001]Au_fcc_//[110]Ge (Fig. 1e). This crystallographic texturing was never directly observed but can be attributed to the preferential orientation of Au crystallites parallel to the germanium <110> direction^14^,^20^. For the samples annealed at 673 K (see Fig. 1d), the EBSD results shows a combination of four possible different configurations of the Au crystallites rotated by 90° (for details see Supplementary Figs S2, S4 and S5, experimental and simulated pole figures and inverse pole figures) with the following two main orientation relationships: case I−(10-11)_tilted by ~8deg_ Au_hcp_//(001)Ge, [2-1-10]Au_hcp_//[110]Ge and case II–(0001)_tilted by ~30deg_ Au_hcp_//(001)Ge, [2-1-12]Au_hcp_//[110]Ge, see schematic in Fig. 1e. The relationship between the average grain size of the Au crystallites’ crystallographic structure and the post annealing temperature, as revealed by EBSD, is presented in Fig. 1f. Here, two temperature regions are clearly visible: 1) For annealing temperatures below T_e_, the crystallites belong to the Au_fcc_ phase, and their size (around 40 nm) is weakly dependent on the annealing temperature; 2) For annealing temperatures above T_e_, Au crystallites containing the Au_hcp_ phase are formed. The size behavior of these crystallites presents a strong temperature dependence increasing from ~40 nm to ~120 nm in the studied temperature range (for details, see Supplementary Fig. S6). In addition, we have checked that the hexagonal form of gold was also present for different Au coverage i.e. 3, 12 and 18 ML as presented in Fig. 1g–i.

The rectangular islands observed below *T*_*e*_ result from a self-assembling process induced by the surface diffusion of Au atoms. On the contrary, above the eutectic temperature, the Au islands form from a liquid AuGe alloy droplets as observed previously by Sutter *et al*.^21^, inferring that the observed polyhedral Au islands originates from a recrystallization process accompanied by a necessary Ge segregation during cooling of the sample to room temperature. As the Au islands possess a defined crystallographic orientation relative to the Ge substrate, the Au/Ge interface has to play an important role for directing the growth and crystallographic orientation of the Au islands. This is different from the observation of randomly oriented Au nanostructures supported by a Ge pedestal when annealing Au clusters deposited on a Ge(111) single crystal^22^. To examine the exact atomic structure of this Au/Ge(001) interface, cross-sectional samples have been analyzed by high-resolution HAADF STEM imaging, where the contrast is proportional to the atomic number (thus, Au appears brighter than Ge). The HAADF STEM images revealed that on the samples annealed at 498 K (below *T*_*e*_), only a small part of the Au island (~1 nm) is submerged into the germanium substrate, as presented in Fig. 2a. A similar effect of Au atoms migrating into the germanium substrate has been observed earlier by Wang *et al*.^23^ and was attributed to the Au/Ge mixing^14^. Moreover, the HAADF STEM image demonstrates, in Fig. 2a, that the epitaxial relationship between the island and the substrate is (101)Au_fcc_//(001)Ge in agreement with the EBSD results presented above.

The possible diffusion of Au atoms into the Ge substrate is further studied using statistical parameter estimation theory24,^25^. Here, the total scattered intensity by each atomic column is computed, as it scales with the average atomic number Z of an atomic column^26^,^27^. By assuming a constant sample thickness, this measure can be used to distinguish between columns containing Au atoms and pure Ge columns. Due to the noise inherent to the experiment, intermixing between the Au and Ge phases is studied atomic plane-by-plane across the interface using two sample t-tests^28^ (see Supplementary Fig. S7 for details). The results presented in Fig. 2b indicate that no Au atoms are present in the Ge substrate, indicating that the interface is perfectly sharp and confirming the non-miscibility of Au in Ge^15^,^23^. For samples annealed above *T*_*e*_, more than half of each of the Au island is buried into the germanium substrate, as shown in Fig. 2c. From HAADF imaging, we determined that the Ge {001} and {111} planes are predominantly interfaced with the buried part of the Au island (for details, see Supplementary Figs S8 and S9). Moreover, for most of the studied Au islands, at the side opposite to their longest edge, a germanium crystalline rim is seen on top of the Ge substrate (Fig. 2c,f). An Au_hcp_ phase, which we will study in details in the following paragraph, is present in the topmost part of the islands. At the bottom of the Au island, the Au_fcc_(011) plane grows epitaxially on a Ge(001) surface, as shown by several HAADF STEM images. The combination of statistical parameter estimation theory with two-sample t-tests shows that in a small region, pure Ge columns are intermixed with columns containing Au. Note that Au atoms are only present in the columns following the *fcc* Au lattice. This leads us to the conclusion that Au atoms are most likely not diffusing into the Ge substrate; thus, excluding the presence of an AuGe alloy, as reported in earlier studies^21^,^29^ (further detailed analysis is available in the Supplementary material, Fig. S7a). Moreover, our experimental findings indicate that the Au islands formed at annealing temperatures lower than *T*_*e*_ can be considered as defect-free single crystals of *fcc* Au, whereas islands formed at higher annealing temperature contain regions of both the Au_hcp_ and Au_fcc_ crystallographic phases clearly resolved by STEM. At the bottom (earlier stage of the crystallization), the Au_fcc_ phase is observed, forming at the Au_fcc_(011)/Ge(001) interface. At the regions facing the Ge(111) planes, the Au_hcp_ phase is observed and grows on the Au_fcc_ {111} planes giving the interfacial relationship (0001)Au_hcp_//(111)Au_fcc_, as seen in Fig. 2c. This growth results in an orientation relationship relative to the Ge substrate as already observed by EBSD, described as (0001)_tilted by ~30deg_ Au_hcp_//(001)Ge, [2-1-12]Au_hcp_//[110]Ge.

Figure 2e shows the radial power spectrum density (PSD) of this Au_hcp_ region. There are two main peaks at 2.442 Å and at 2.233 Å, which can be uniquely identified as the (0002) and (10–11) reflections of the Au_hcp_ phase^8^. The derived experimental lattice constants for Au_hcp_ are a = 2.90 Å and c = 4.88 Å, which are in good agreement with other experimental^8^ and theoretical values^13^. STEM images show that the Au_hcp_ phase is always present at the top part of the island, which explains the absence of Au_fcc_ phase in the EBSD measurements presented above. In light of the HAADF STEM images, models of the interfaces Au_fcc_(011)/Ge(001) and (0001)Au_hcp_//(111)Au_fcc_ have been analyzed. At the bottom of the islands, the Au_fcc_(011) plane is growing epitaxial to a Ge(001) surface. In the plane of the interface, the Au_fcc_(100) and the Ge(110) lattice planes are in good agreement (4.078 Å/4.000 Å = 1.0195), and the two structures match well in this projection (see Fig. 3a). The Au_hcp_ and Au_fcc_ structures represent two different stacking sequences of close packing; hence, the interface between (0001)Au_hcp_ and (111)Au_fcc_ will be in perfect registry (see Fig. 3b). In contrast to the free standing Au_*hcp*_ square sheets studied by Huang *et al*.^8^, the Au_*hcp*_ nanostructures formed on Ge(001) are stable under electron irradiation. Furthermore, it has been found that both the annealing temperature and cooling rate are important in order to control the final phase content of Au nanostructures. Here, two series of measurements have been used: annealing at 673 K and 773 K and then cooling down to RT with constant rates of 0.1 K/min, 15 K/min and 700 K/min. The analysis of samples annealed at 673 K shows the existence of three different regions in the island structures: a pure unfaulted Au_hcp_ phase; a hexagonal intergrowth domain consisting of a mixture of different stacking sequences which could be attributed to the pure Au_hcp_ and Au_fcc_ atomic planes simultanously; and a pure Au_fcc_ phase close to the germanium substrate. For samples annealed at 773 K, significantly less regions having the Au_hcp_ phase are observed in favor of an intergrowth domain content (see Figs S10 and S11). The structure of the pure unfaulted Au_hcp_ phase can be seen in Fig. 4a. The interface between the intergrowth domain and the pure Au_hcp_ is presented in Fig. 4b. The formed Au nanostructures were free of any germanium content within the precision of the performed EELS spectroscopy measurements Fig. 4c–e. Furthermore, the cooling rate is important in order to improve the formation of the pure Au_fcc_ and Au_hcp_ phases. At a fast cooling rate (i.e., 700 K/min), the domains of intergrowth between Au_fcc_ and Au_hcp_ are significantly increased compared to slower cooling, where the pure phase domains are larger, dictating this phenomenon to be thermodynamically controlled.

According to the experimental findings discussed here, a model explaining the formation of the observed half-buried Au islands on the Ge(001) substrate can be proposed, as presented in Fig. 5a–d. It is separated into 4 steps.

- At the beginning of the annealing process, the deposited Au atoms aggregate into clusters due to surface diffusion of Au atoms on an Au rich surface and particle coarsening^19^. When the temperature exceeds the eutectic temperature of the system, a liquid Au-Ge droplet is formed by incorporation of Ge atoms from the substrate into the hot Au clusters, as described previously in nanowires^12^. According to the Au/Ge phase diagram, the concentration of germanium in the Au-Ge droplets is dependent on the annealing temperature^15^. In the process of germanium incorporation into the Au droplet, some truncated pyramid-like germanium pits are formed, similarly to ones observed in the metal-assisted etching process^30^, with the lowest surface energy Ge {001} facets at the bottom and the slightly more energetic Ge {111} planes on the pit’s sides^31^.
- During the slow cooling of the sample (0.1–15 K/min), the Au-Ge droplet undergoes recrystallization, beginning with the growth of Au in its preferred Au_fcc_ structure along the [011] direction on the bottom of the pit, since the Ge(001) and Au(011) planes are in registry (a misfit of only 1.94%) as seen in Fig. 3a. The developing Au_fcc_ crystallite grows with little twinning, and its crystallization front is along its {111} facets, which are energetically favorable (i.e., they have the lowest surface energy)^32^.
- The formation of these Au_fcc_ crystallites leads to the germanium enrichment of the remaining liquid, so that the metastable hexagonal β-AuGe alloy is formed, similarly as directly first seen in the case of Au nanostructures supported on germanium nanorods by Marshall *et al*.^12^ and accordingly to Okamoto *et al*.^15^, Sutter *et al*.^21^ and Hajjar *et al*.^22^.
- Upon further cooling, the metastable alloy separates into the Au_hcp_ phase and pure Ge due to the easily diffused Ge atoms from the β-AuGe alloy, as was seen in the case of TEM *in-situ* studies on nanowires^12^. The formed Au nanostructures are free of any germanium content as checked by the EELS measurements Fig. 4c–e. The excess Ge from the alloy segregates at the opposite of the Au_fcc_ crystallite and forms a crystalline rim at the alloy/Ge interface, as shown in Fig. 2c,f. Moreover, the excess Ge diffuses along the reconstructed substrate surface and extends the atomic terraces. This is evidenced by a drop in the intensity of RHEED spots during the cooling of the sample in the range from ~463 K down to ~433 K, see Fig. 4f. Our findings are consistent with the experimentally observed germanium out-diffusion from Au-Ge metastable phase which begins at ~448K^12^ and the decay takes ~5 min^15^.

This scenario holds for lower cooling rates of 0.1–15 K/min, which indicates that this mechanism is thermodynamically stable and that our system is at a constant thermodynamic equilibrium during the cooling process. In the case of Au droplets on germanium nanorods, with finite amount of germanium available in the system as limited by the size of the germanium nanorod, the slow cooling resulted in the formation of the single crystalline segments of metastable γ-AuGe alloy^21^. However, for very fast cooling (i.e., 700 K/min), the pure Au_hcp_ phase is not formed at the expense of an intergrowth of Au_fcc_ and Au_hcp_. In contrast to fast cooling of the Au droplets on germanium nanorods presented by Marshall *et al*.^12^, Au_hcp_ was observed. This might be related to the modification of the Ge diffusion kinetics due to the change of diffusion regime from a finite to an infinite Ge concentration at the source. In fact, the nanoscale Au-droplet phase diagram (valid for the nanorod system), with finite amount of germanium limited by size, deviated significantly from the phase diagram for bulk phase, as shown by E. Sutter and P. Sutter^33^,^34^. In our case, for an infinite amount of germanium available, the presence of the β-AuGe alloy, which is a precursor for the formation of pure Au_hcp_ (as suggested by the Au/Ge phase diagram^15^), is responsible for this change of regime. As the pure Au_hcp_ phase is formed only when the annealing is above *T*_*e*_ but below 773 K, we suggest that the formation of the β-AuGe alloy depends on the initial germanium concentration in the hot droplet. Considering our observations and the Au/Ge phase diagram^15^, the germanium content in the Au-Ge droplet should be higher than 28 at. % (at 663 K) but lower than 38 at. % (at 773 K) to form pure Au_hcp_. This is summarized in the derived Au/Ge phase diagram for the self-assembled Au nanostructures on the Ge(001) surface presented in Fig. 5e.

In summary, the formation of crystalline Au nanostructures on a germanium substrate during a thermally induced self-assembly process of 6 ML Au deposited on a Ge(001) surface was investigated. The Au islands change from a rectangular shape made of the Au_fcc_ phase to a polyhedral shape belonging mainly to the Au_hcp_ phase while the annealing temperature crosses ~634 K; this temperature corresponds to the eutectic temperature for the Au-Ge system. The formation of the *hcp* phase of Au was found to result from the decay of the β-AuGe metastable alloy when it is cooled down at a sufficiently slow rate (15 K/min or lower). We can conclude that the Au crystallization starts from one side of the germanium pit, i.e., the Ge(111)/Ge(001) plane corner, into the favorable Au_fcc_ phase with {111} facets followed by the formation of the β-AuGe alloy under the influence of a depletion of Au from this crystallizing liquid. Finally, at a temperature of ~463 K, the metastable β-AuGe alloy separates into the Ge rim surrounding the island and the Au_hcp_ phase on top of the Au_fcc_ {111} facets. Confronting our observations to the known Au/Ge phase diagram, the germanium content in the Au-Ge droplet should be higher than 28 at. % (at 663 K) but lower than 38 at. % (at 773 K) to form pure Au_hcp_. The presented simple technology of Au_hcp_ growth will enable the new development in the fabrication of defined templates of this very rare phase of Au and will enable investigation and development of new devices based on *hcp* gold.

## Methods

### Au/Ge sample preparation

The samples were prepared in a UHV MBE system at Institute of Physics Jagiellonian University, Krakow, Poland. The Ge(001) substrate surfaces were cleaned by cycles of low-energy ion beam bombardment and annealing in order to achieve atomically flat terraces. Deposition of 6 ML of Au by the Molecular Beam Epitaxy with 0.12 ML/min resulted in formation of a crystalline, continuous Au overlayer as verified by RHEED and LEED. Just after the deposition, the 6 ML Au/Ge(001) samples were post-annealed in UHV conditions (10^−10^ mbar) to temperatures ranging from 498 K to 773 K, for which the initial continuous Au layer can reform into nanoscale Au islands. The temperature was increased with constant rate of 10 K/min and decreased in the cooling process with a rate of 15 K/min. Then, the samples were transferred *in-situ* by means of a UHV transfer system from a UHV MBE system to the SEM chamber (1*10^−6^ mbar).

### Sample characterization

**Figure 1:**
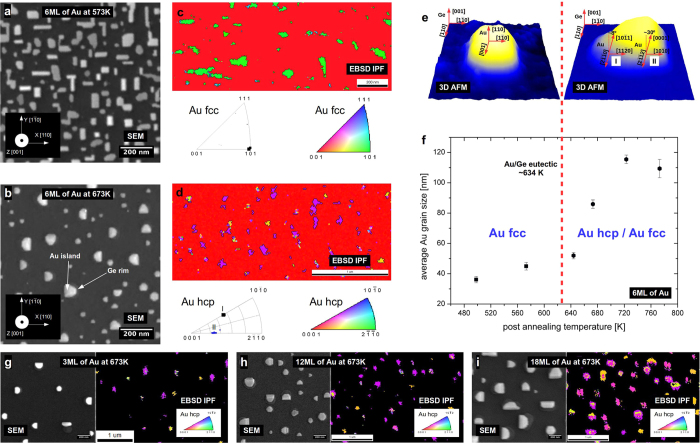
Morphology of the Au nanostructures on a Ge(001) surface formed after annealing. (**a**,**b**) HR-SEM micrographs for post annealing of 6 ML Au/Ge(001) at 573 K (below Au/Ge eutectic) and 673 K (above Au/Ge eutectic); germanium substrate crystallographic directions are indicated. (**c,d**) corresponding EBSD Inverse Pole Figures (Au fcc and Au hcp phase respectively) in the Z direction. (**e)** schematic of orientation relationships for Au nanostructures below and above Au/Ge eutectic. (**f)** Average Au crystalline grain size as a function of the post annealing temperature. Exemplary SEM micrographs and EBSD IPF maps for post annealing at 673 K (above Au/Ge eutectic) for 3 ML (**g),** 12 ML (**h)** and 18 ML of Au (**g)**. In all cases the hexagonal phase of Au was present. For all experiments, the temperature during the cooling down of the sample was decreased at rate of 15 K/min.

The Au/Ge(001) samples were characterized by SEM (FEI Quanta 3D FEG) equipped with a EBSD system (EDAX TSL DigiView camera) at the Institute of Physics Jagiellonian University, Krakow, Poland, which allows structural examination from the near surface of the sample (for Au, it is of a few nanometers). The EBSD diffraction patterns were collected at a 70° sample tilt at 15 keV beam energy. The diffraction patterns were automatically indexed by EDAX TSL OIM DC 7.2.1 software, according to the Au fcc phase and the Au hcp phase. The software algorithm during indexing selects the best matching phase automatically^35^,^36^. The sample geometry was chosen in a such a way that X, Y and Z are parallel to the [110], [1–10] and [001] crystallographic directions of the Ge single crystal substrate, respectively, as indicated in Fig. 1(a,b). IPF maps were subject to two clean-up algorithm procedures to ensure that reliable data were displayed, where grain CI standardization was applied and neighbor CI correlation with CI (0.4). The analysis of the experimental EBSD pole figures and inverse pole figures resulted in the derivation of the epitaxial relations for the formed nanostructures. The Au grain size was calculated from the EBSD data as an average grain feret diameter. For the 498 K sample, the Au grain size was calculated from the STEM cross section measurements. Samples were also investigated with a 5500 Agilent Atomic Force Microscope at ambient conditions. In order to study the internal structure of the Au/Ge(001), cross section samples were prepared using the FIB technique. At first, a protective carbon layer was deposited on the surface inside the SEM before an exposure to the atmosphere to avoid any change to the native structure of the samples. After this, a Pt protection layer was deposited first by e-beam deposition and then by ion-beam deposition to ensure minimal damage to the surface of the sample.

High Angle Annular Dark Field (HAADF) imaging was performed in a Probe Corrected Scanning Transmission Electron Microscope (STEM) FEI Titan at the University of Antwerp, Belgium, operated at 300 kV. This imaging mode provides structural images where intensities are proportional to both the thickness and mean atomic number *Z*. Statistical parameter estimation theory was used to calculate the total scattered intensity of the individual projected columns^24^,^25^. A linear scaling of these intensities with the atomic number Z was assumed to quantify the chemical structure of the Au/Ge interface^26^,^27^. A statistical analysis by two-sample t-test^28^ using the mean scattered intensity of the different columns in each atomic layers was performed to determine which layers have columns containing Au atoms.

To investigate the presence of germanium in the Au nanostructures, EELS measurements were performed. EELS was preferred to EDX due to the possible contribution of secondary fluorescence effects of the highly energetic Au K line giving rise to an artificial germanium signal in the Au. The EELS spectra were acquired at 300 kV with a convergence angle of 20 mrad and a collection angle of 100mrad. The spectra were acquired in Dual EELS with the same exposure time of 0.1 s/pixel and a dispersion of 0.5 eV/pixel. The spectra were then spliced together using the DM routine. The spectra obtained for region 1 and region 2 were power-law background subtracted in the region from 100 to 1150 eV.

## Additional Information

**How to cite this article**: Jany, B. R. *et al*. Controlled growth of hexagonal gold nanostructures during thermally induced self-assembling on Ge(001) surface. *Sci. Rep.* **7**, 42420; doi: 10.1038/srep42420 (2017).

**Publisher's note:** Springer Nature remains neutral with regard to jurisdictional claims in published maps and institutional affiliations.

## Supplementary Material

Supplementary Information

## Figures and Tables

**Figure 2:**
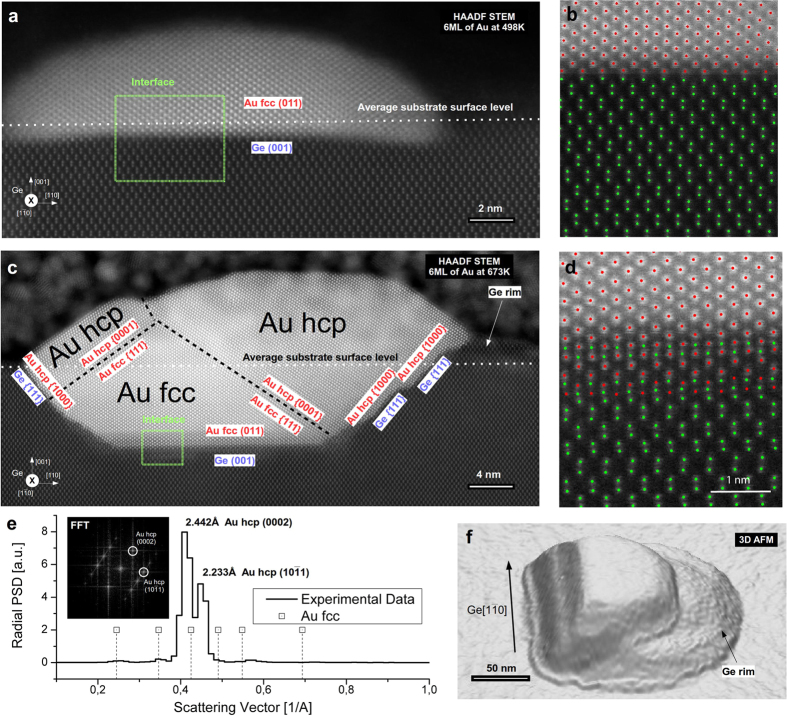
Atomically resolved STEM-HAADF images of the Au nanostructures formed on a Ge(001) surface after annealing the sample: **(a)** at 498 K, below the eutectic temperature of Au/Ge and (**c)** at 673 K, above the eutectic temperature. (**b**,**d)** The Au(011)/Ge(001) interface region from the zones marked in (a) and (c). Columns containing only Ge atoms are indicated in green, and columns containing Au atoms are indicated in red. (**e)** Radial power spectrum density (PSD) of the Au_hcp_ region of the Au island; atomic spacing corresponding to Au_fcc_ and Au_hcp_ are indicated. The two major peaks are identified as (0002) and (10–11) of Au hcp phase. Insert: FFT of the hcp region. (**f)** tapping-mode AFM 3D topography of the Au island.

**Figure 3:**
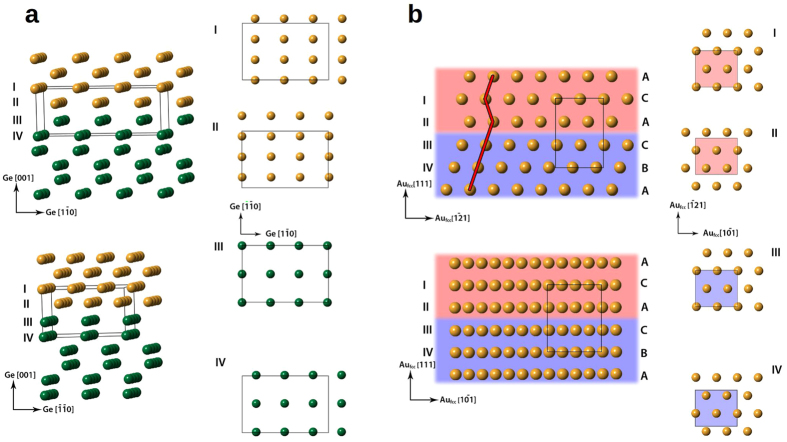
Model of the interfaces derived from atomically resolved STEM-HAADF measurements. (**a)** atomistic model of the interface between Au_fcc_ (yellow) and Ge(001) (green)viewed along Ge[-1-10] zone axis (top) and Ge[1–10] zone axis (bottom) on the right four sections of marked atomic planes (I, II, III, IV) at the interface. Ge bulk crystallographic directions indicated; (**b)** atomistic model of the interface between Au_hcp_ (on red) and Au_fcc_ (on blue) viewed along the Au_fcc_ [10−1] zone axis (top) and the Au_fcc_ [1–21] zone axis (bottom) on the right four sections of marked atomic planes (I, II, III, IV). Au_fcc_ crystallographic directions are indicated.

**Figure 4:**
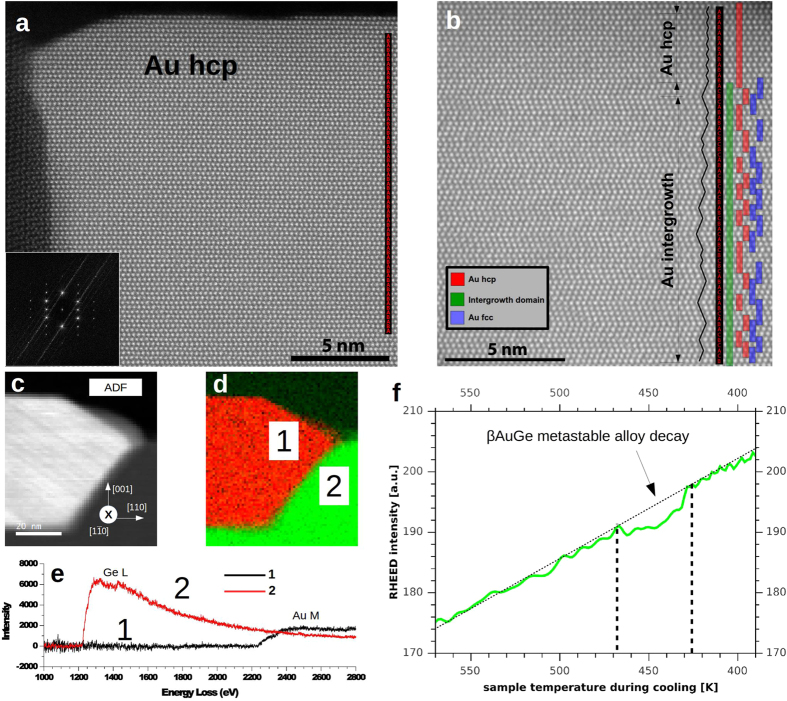
Atomically resolved HAADF STEM of pure Au hcp phase (**a)** together with FFT of that region. Au intergrowth region (**b)** made of two Au domains fcc and hcp. A black guide line is added following the stacking of the horizontal layers. The stacking sequence is labeled with the letters A, B and C in order to define the stacking sequence. Results of the EELS spectroscopy measurements of the Au islands: ADF-STEM of the examined area (**c)** and corresponding EELS Ge L_2,3_ (green) and Au M_4,5_ (red) map (**d)**. In (**e)** EELS spectrum is presented from area 1 (the Au island) and area 2 (germanium). It is seen that within the precision of the EELS spectroscopy the Au island is free of any germanium. (**f)** Change of the RHEED intensity, recorded during sample cooling, after post annealing of 6 ML Au/Ge(001) to temperature higher then Au/Ge eutectic. Monotonic increase of the intensities is seen for all type of surface reconstruction spots. In the temperature range of 470 K–423 K a drop of the intensity occurs, which takes ~8 min, later the intensities increase monotonically in the same way. The observed drop of RHEED intensity is assigned to the decay of the beta-Au-Ge metastable phase leading to formation of Au hcp and pure germanium.

**Figure 5:**
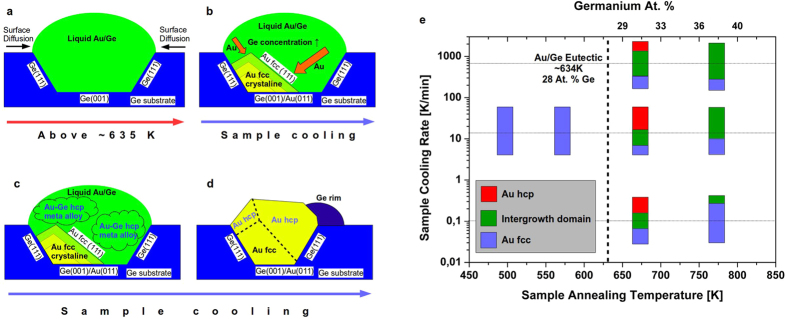
Mechanism of the Au hcp islands formation after annealing the sample at temperatures exceeding the Au/Ge eutectic temperature (above ~634 K). (**a)** deposited Au aggregation due to the surface diffusion and formation of Au/Ge liquid droplet. Germanium substrate etching and formation of truncated inverted pyramidal pits; (**b)** cooling the sample–Au recrystallization from the liquid into the fcc phase with simultaneous Ge enhancement in the remaining liquid; (**c)** formation of βAuGe hcp alloy; (**d)** formation of Au hcp phase and Ge rim from the βAuGe hcp alloy decay at sample temperature of ~463 K. (**e)** Experimentally derived Au/Ge phase diagram for self-assembled Au nanostructures on a Ge(001) surface, sample cooling rate as a function of sample annealing temperature and germanium content in Au/Ge liquid The relative sizes of Au_hcp_(red), intergrowth domain(green) and Au_fcc_(blue) regions of the island after recrystallization of the liquid are marked. When changing the sample annealing temperature and sample cooling rate, the Au_hcp_ relative sizes change.
