# Supplementary material for: Controlled growth of hexagonal gold nanostructures during thermally induced self-assembling on Ge(001) surface
Source: Sci Rep. 2017 Feb 14;7:42420. doi: 10.1038/srep42420 (PMC5307968; doi:10.1038/srep42420)
Supplement: Supplementary Information [file srep42420-s1.pdf]

Supplementary Materials

Controlled growth of hexagonal gold nanostructures during thermally induced self-assembling on Ge(001) surface

B.R. Jany[a),*], N. Gauquelin[b),*], T. Willhammar[b)], M. Nikiel[a)], K.H.W. van den Bos[b)], A. Janas[a)], K. Szajna[a)], J. Verbeeck[b)], S. Van Aert[b)], G. Van Tendeloo[b)], F. Krok[a)]

[a)] Marian Smoluchowski Institute of Physics Jagiellonian University, Lojasiewicza 11, PL-30348 Krakow, Poland

[b)] EMAT University of Antwerp, Groenenborgerlaan 171, BE-2020 Antwerp, Belgium

*equally contributing authors

Correspondence to benedykt.jany@uj.edu.pl (Benedykt R. Jany) and Nicolas.Gauquelin@uantwerpen.be (Gauquelin Nicolas)

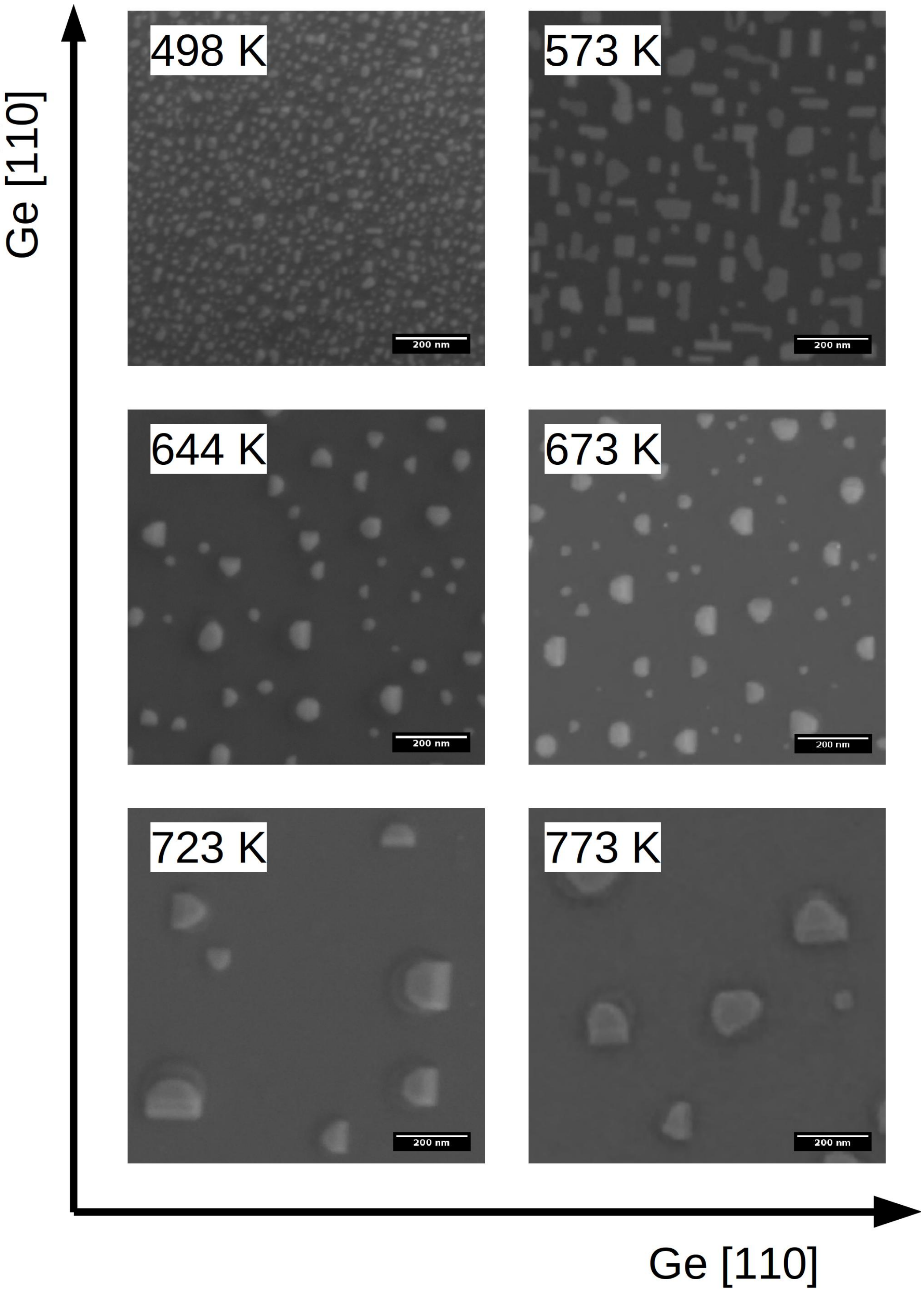

Figure S1: SEM shape evolution of the Au nanostructures on Ge(001) surface upon thermally organization of deposited 6 ML of Au on Ge(001). Ge crystallographic directions indicated.

EBSD Pole Figure and Inverse Pole Figure studies

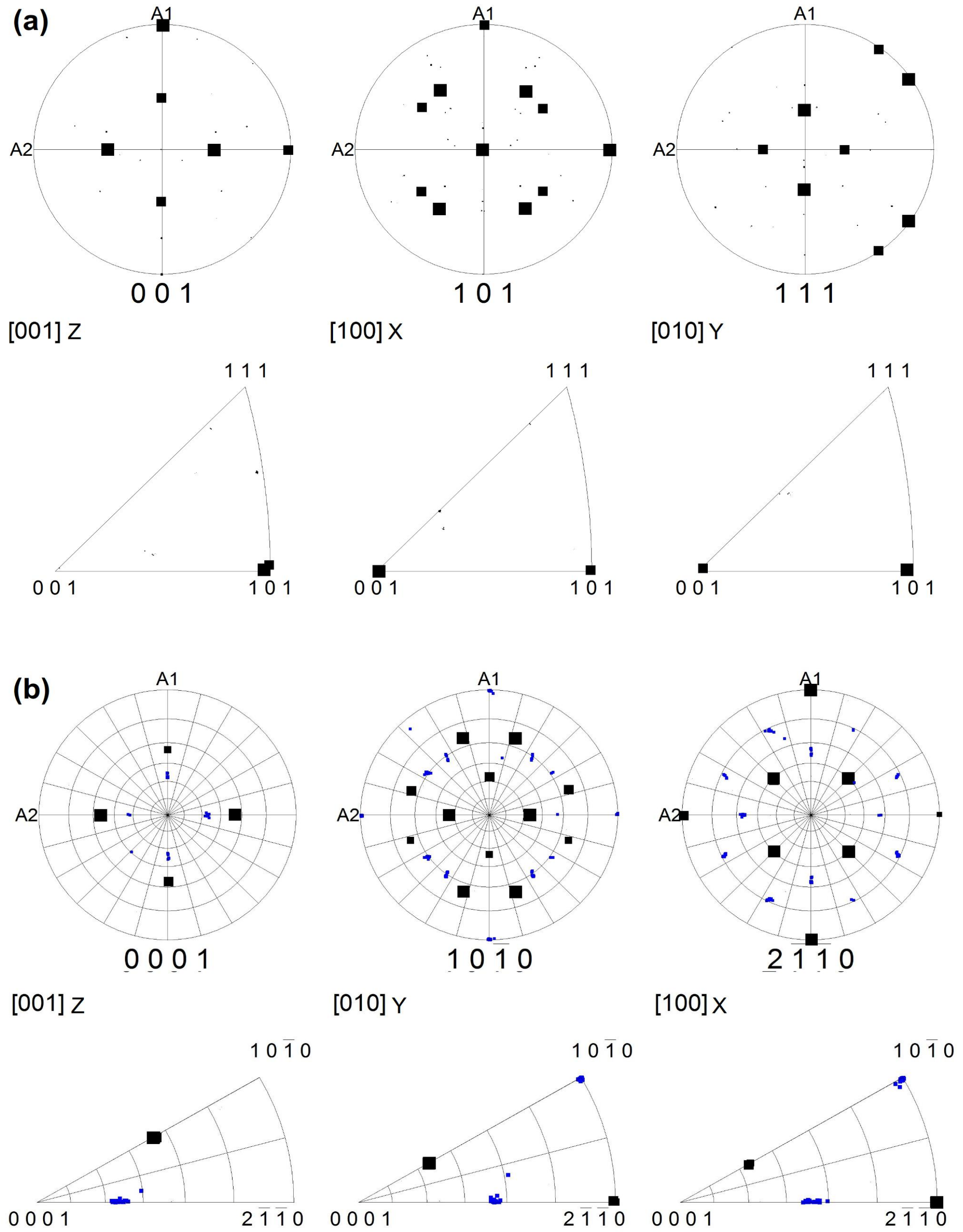

Figure S2: Experimental Pole Figure and Inverse Pole Figures for Au nanostructures formed on Ge(001) surface upon thermally organization of deposited 6 ML of Au annealed a) at 573K (below Au/Ge eutectic) and b) at 673K (above Au/Ge eutectic), at 673K two orientations are visible I(black) and II(blue).

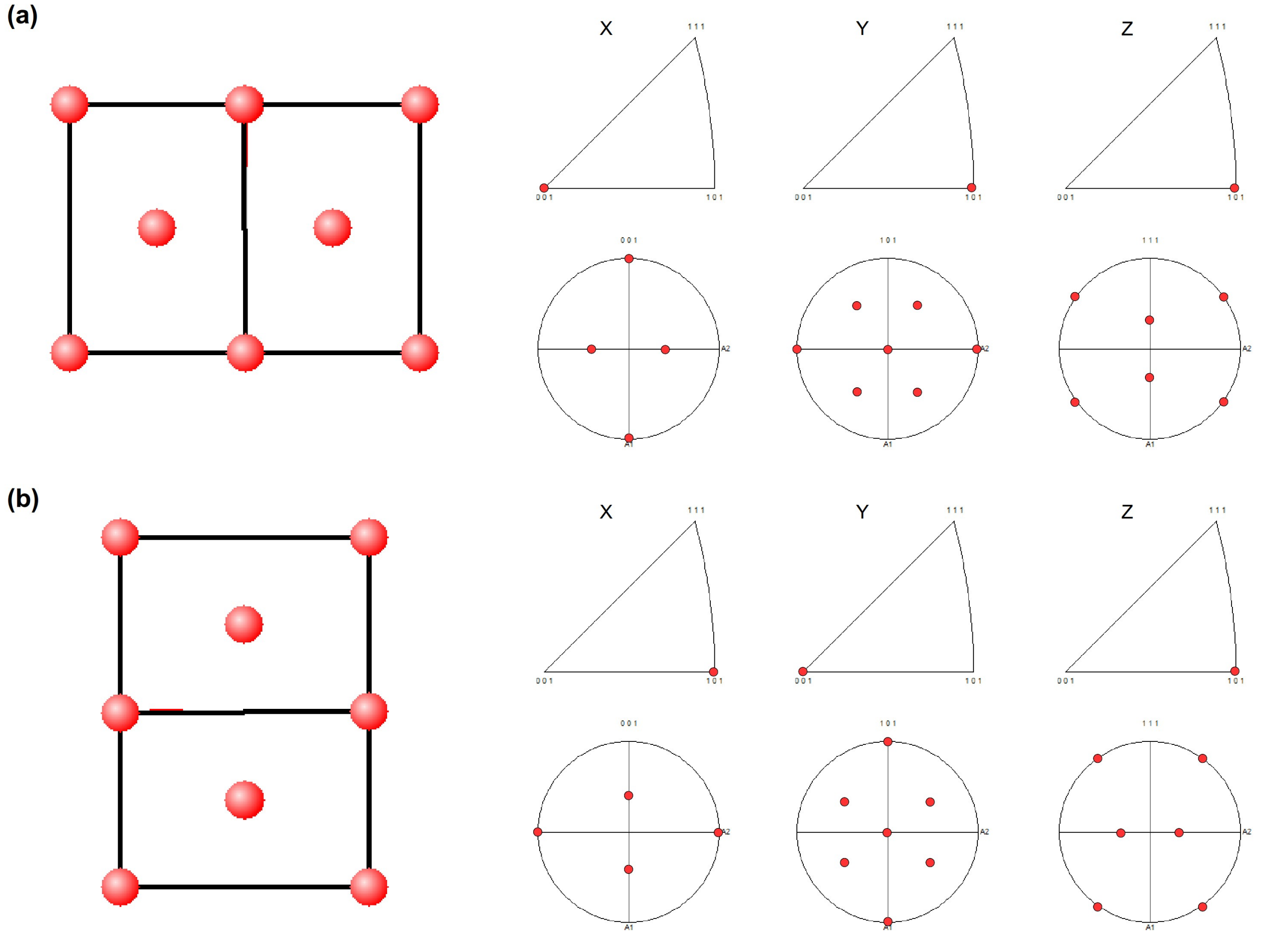

Figure S3: Simulation of Inverse Pole Figure (IPF) along X, Y and Z direction and Pole Figures (PF) (001, 101, 111) for two configuration of Au fcc crystallites: (a) (011) surface oriented along Z direction, (b) (011) surface oriented along Z direction, rotated by 90 deg in-plane.

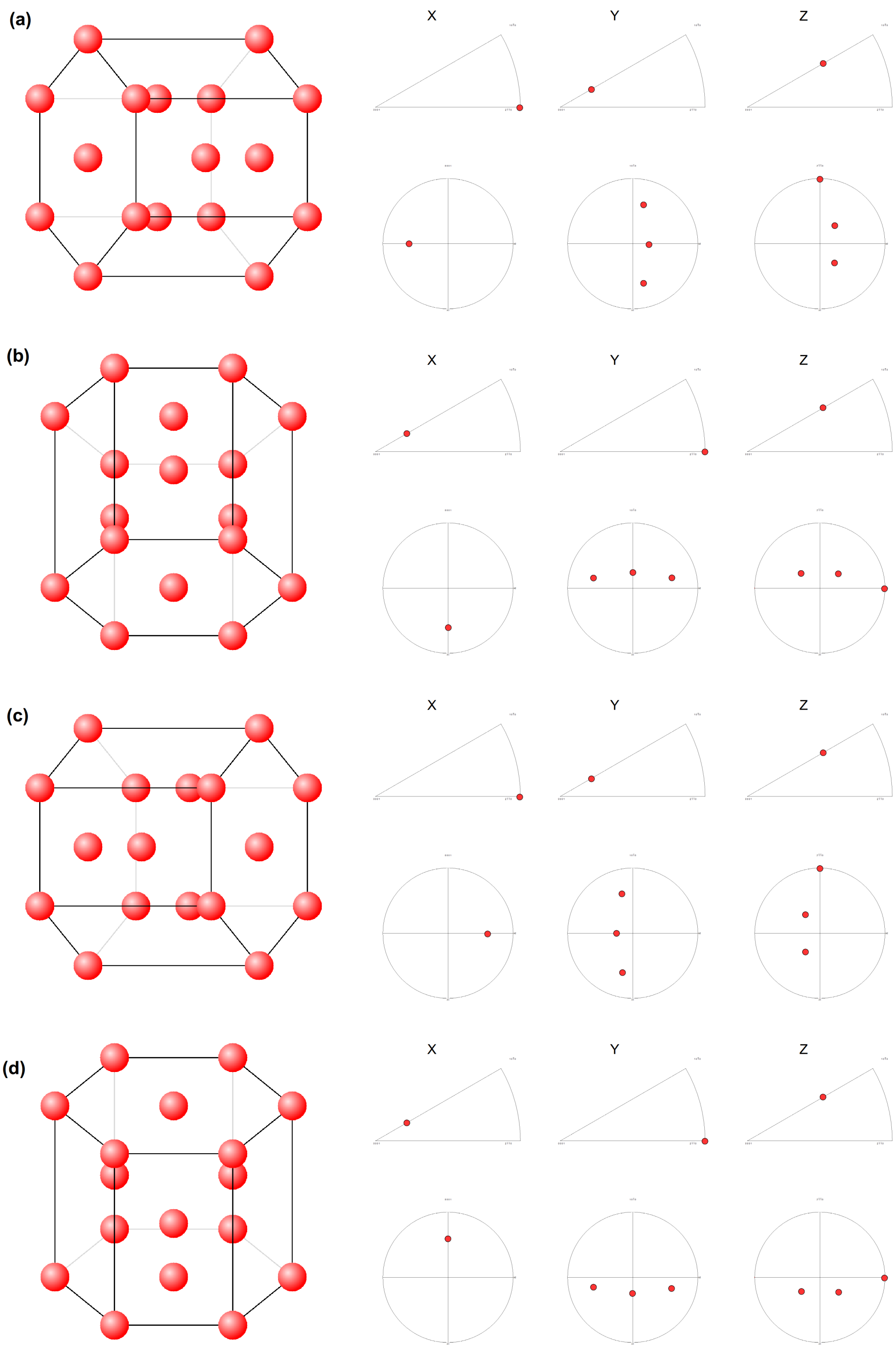

Figure S4: Simulation of Inverse Pole Figure (IPF) along X, Y and Z direction and Pole Figures (PF) (0001, 1010, 2110) for four configuration of Au hcp crystallites rotated in plane by: (a) 0 deg, (b) 90 deg, (c) 180 deg, (d) 270 deg; for the case of orientation I(black) of Au hcp crystallites, see Fig. S2(b).

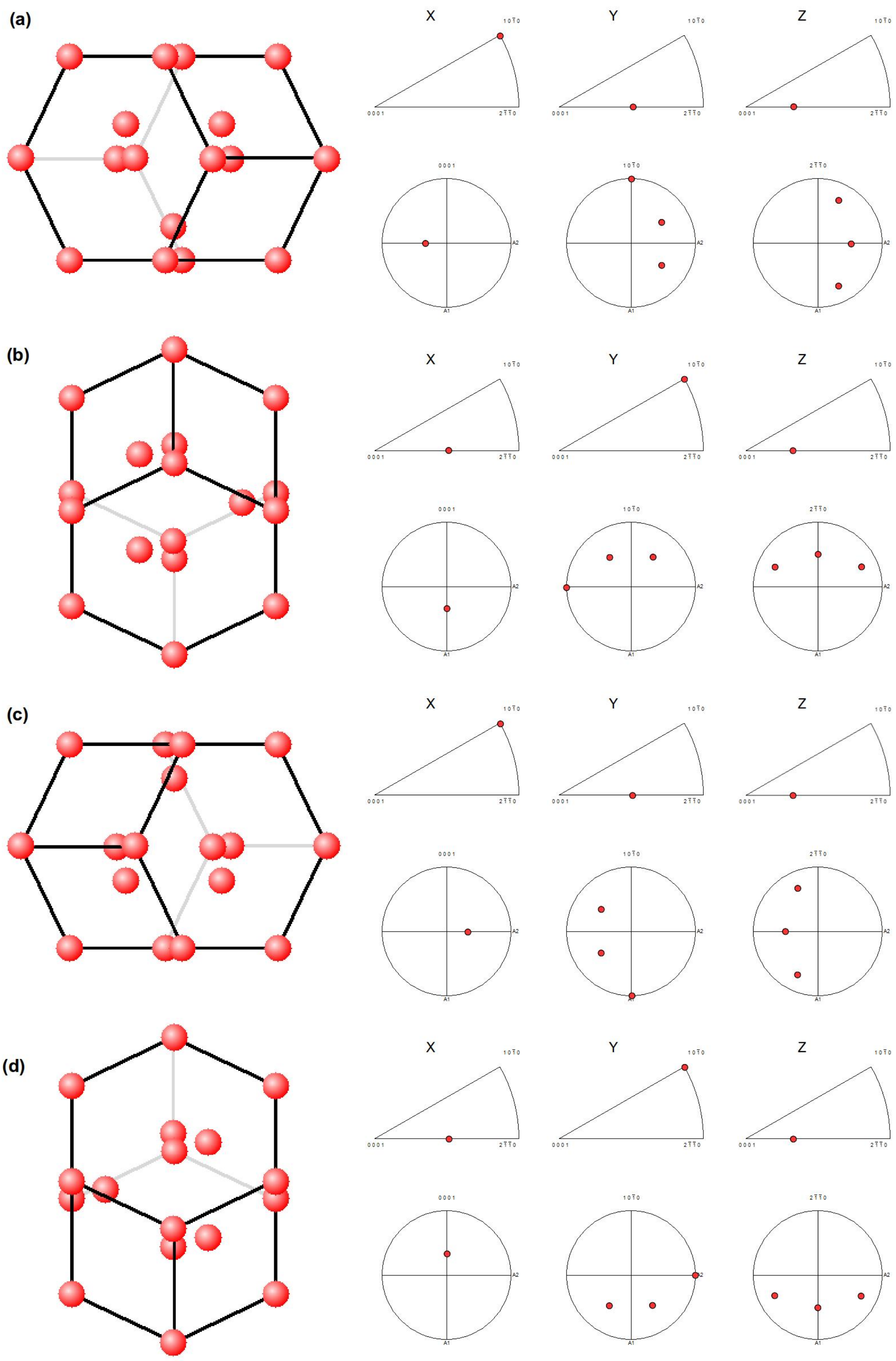

Figure S5: Simulation of Inverse Pole Figure (IPF) along X, Y and Z direction and Pole Figures (PF) (0001, 1010, 2110) for four configuration of Au hcp crystallites rotated in plane by: (a) 0 deg, (b) 90 deg, (c) 180 deg, (d) 270 deg; for the case of orientation II(blue) of Au hcp crystallites, see Fig. S2(b).

EBSD Au grain size distribution

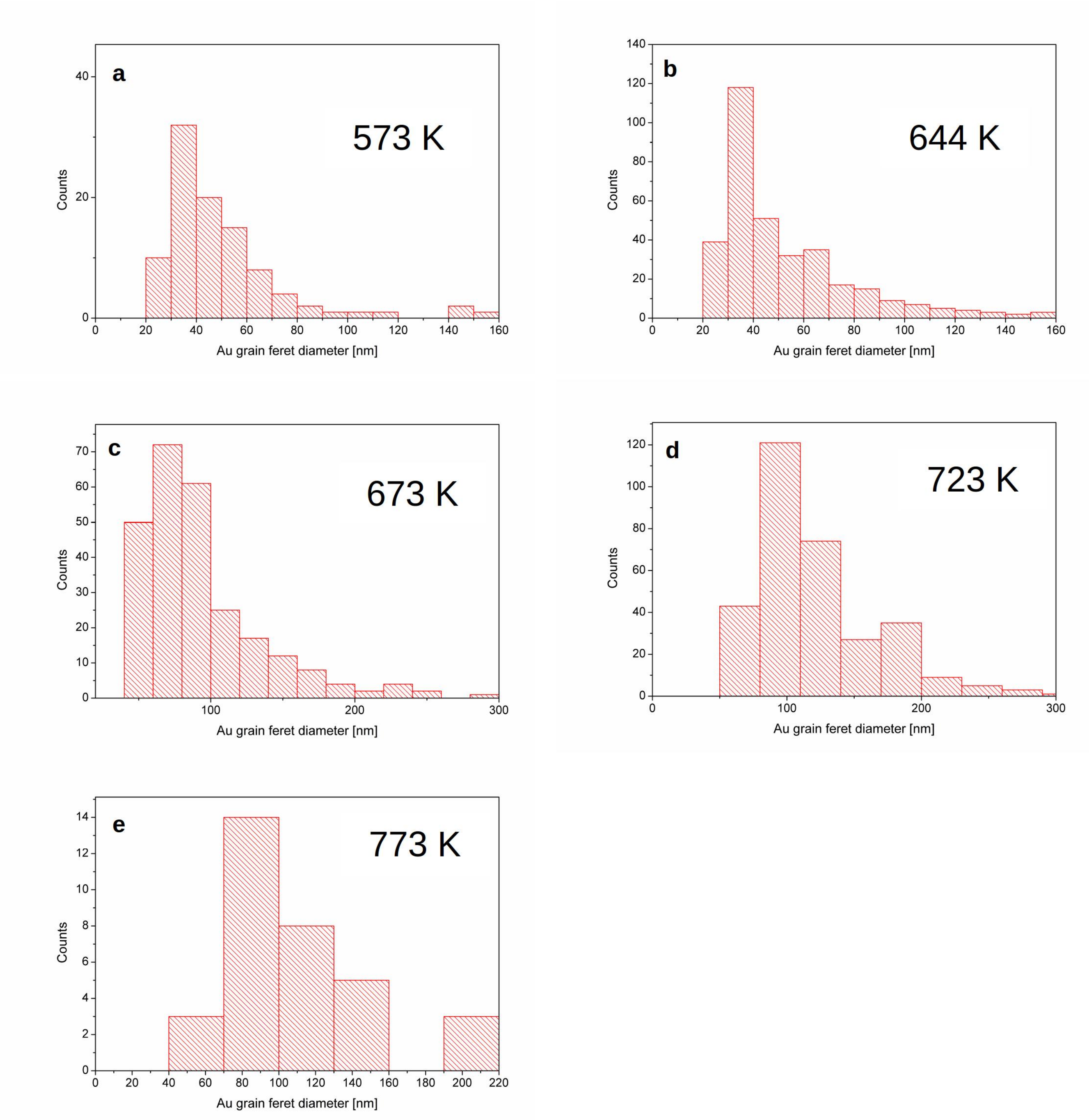

Figure S6: Au grain size distribution calculated from the collected EBSD data as a feret diameter, for different post annealing temperatures of the 6ML Au/Ge(001) samples. For the sample post annealed to 498K the Au grain size was calculated from the measurements of 10 nanoislands from TEM cross sections, this gave average size of 36.1 ± 2.1 nm.

Statistical analysis of the Au/Ge interface

In order to distinguish columns containing Au atoms from pure Ge columns, the total scattered intensity of each atomic column is measured by statistical parameter estimation theory [6,7]. When assuming a constant sample thickness these scattered intensities scale with the average atomic number Z of an atomic column. By using two sample t-tests, scattered intensities are atomic planes by atomic planes compared to scattered intensities of a reference area (the brown areas in Figure S5) in which all columns are considered to be pure Ge columns. Therefore, the reference area was chosen at a relatively large distance from the interface. To visualize the analysis, the yellow region in figures S5b and S5d indicates the range of scattered intensities in which atomic planes contain only pure Ge columns. The results in Figure S5a and S5b indicate that only in one atomic plane columns contain Au atoms, indicating that for Au islands formed by annealing below the eutectic temperature the interface is sharp. In figure S5c and S5d it is shown that in only two atomic planes columns contain Au atoms. Note that the columns of these atomic planes are positioned both on the Ge and the Au lattice. Since no Au atoms are observed in the other atomic planes, diffusion of Au atoms in Ge substrate seems to be negligible. Therefore, it is assumed that also for Au islands formed by annealing above the eutectic temperature a sharp interface is present. This is also confirmed by the rapid decrease of the scattered intensities following the Au lattice towards the interface, as is shown in Figure S5f.

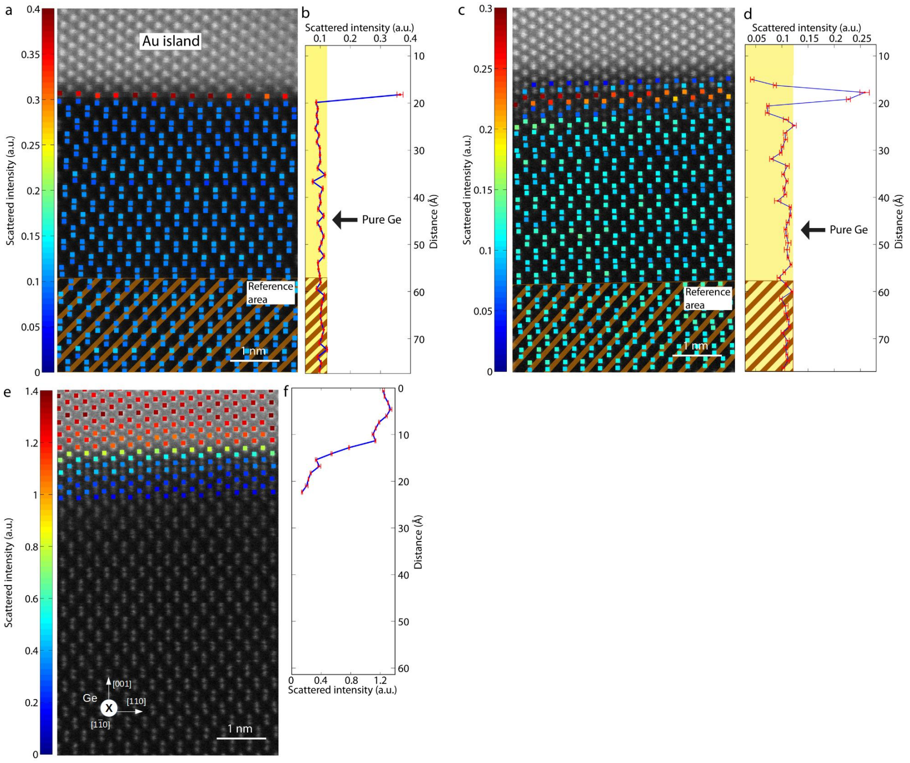

Figure S7: The scattered intensities of the columns following the Ge lattice of Figure 2b (a) and Figure 2d (c). The mean scattered intensity of the columns along the horizontal [110] direction of Figure S5a (b) and S5c (d). Two-sample t-tests indicate that atomic planes having columns with a mean scattered intensity within the yellow region do not contain Au atoms. Here, the brown indicated areas are chosen as a reference, in which only pure Ge columns are present. (e) The scattered intensities of the columns following the Au lattice of Figure 2d. (f) The mean scattered intensity of the columns along the horizontal (011) direction.

Crystallography of Au nanostructures

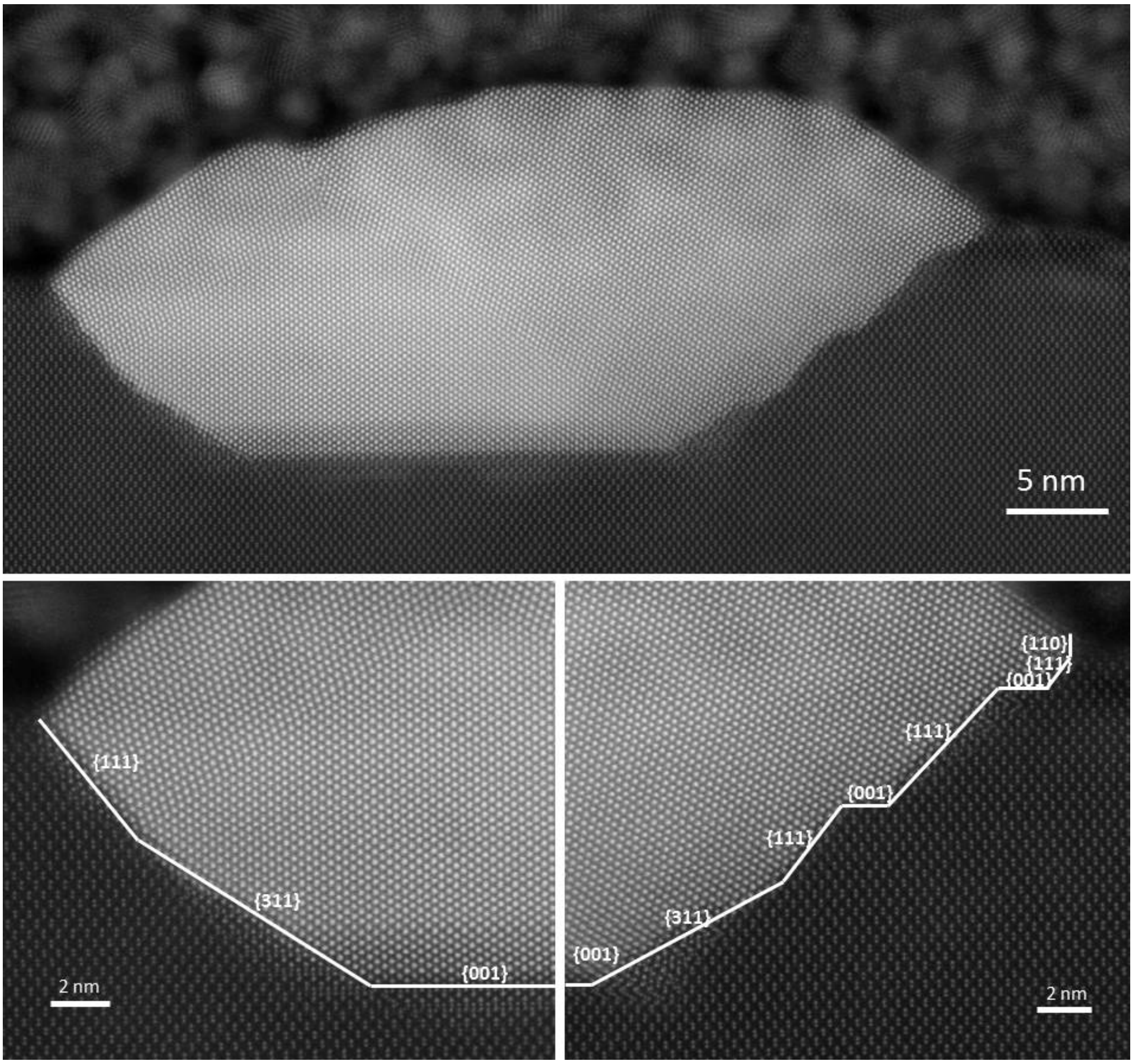

Figure S8: Atomically resolved STEM-HAADF image of the buried Au island exhibiting the facets orientations along the Germanium {001}, {111} and {311} planes.

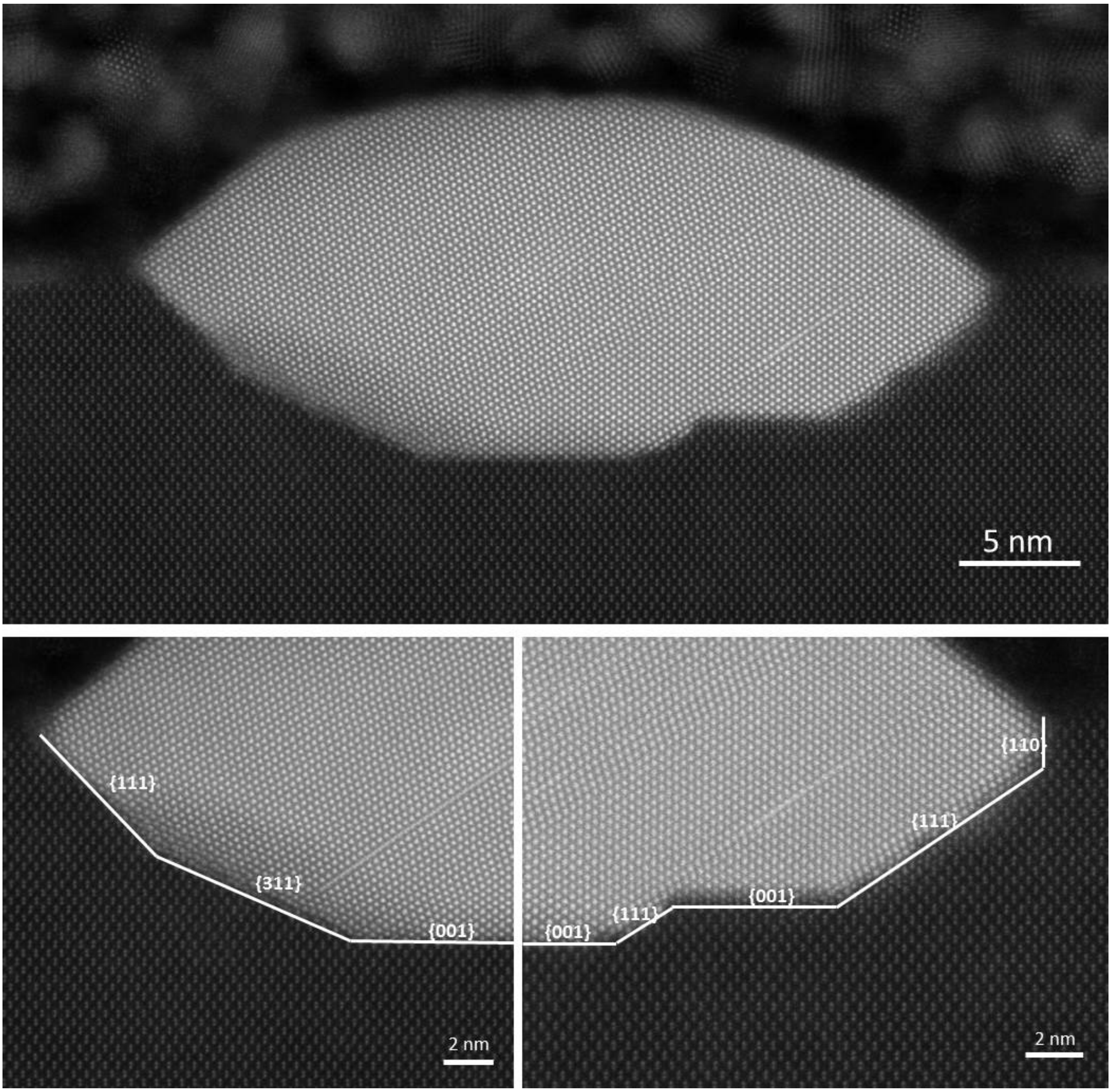

Figure S9: Atomically resolved STEM-HAADF image of the buried Au island exhibiting the facets orientations along the Germanium {001}, {111} and {311} planes.

Effects of sample cooling rate and annealing temperature

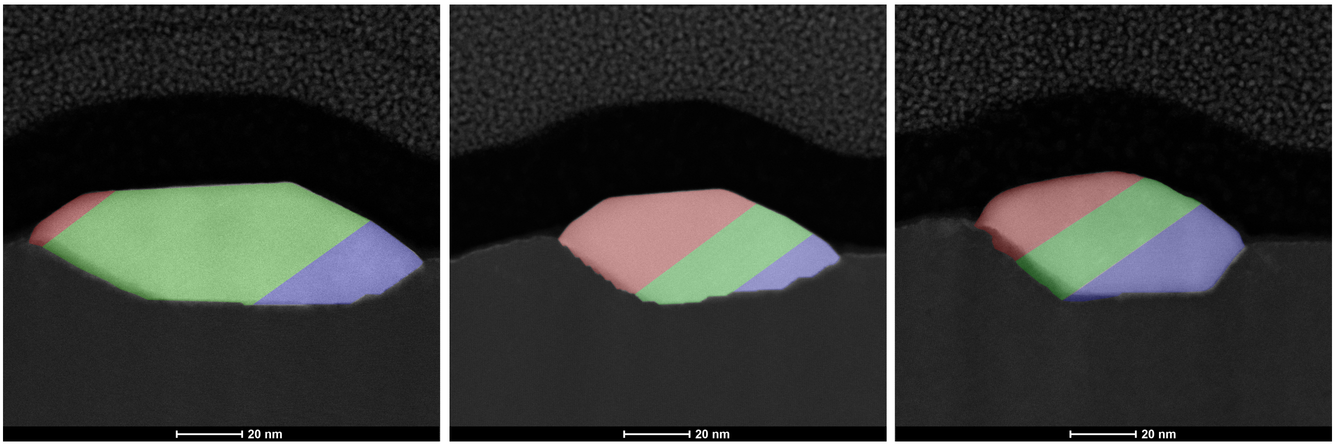

Figure S10: Three representative HAADF-STEM images of FIB sectioned lamellas from the sample annealed at 673 K for three different cooling rates, (a) 700 K/min, (b)15 K/min (c)0.1 K/min, the islands are viewed along the [110]Au$_{fcc}$/[100]Au$_{hcp}$ direction. In all three samples Au$_{fcc}$ phase is growing at the bottom right corner (blue). (a) In the sample prepared with a fast cooling rate a larger domain of intergrowth between Au$_{fcc}$ and Au$_{hcp}$ can be observed (green) and just a limited growth of Au$_{hcp}$ (red). The medium and slow cooling rates forms larger domains of pure Au$_{hcp}$ phase with a limited amount of faulted stacking.

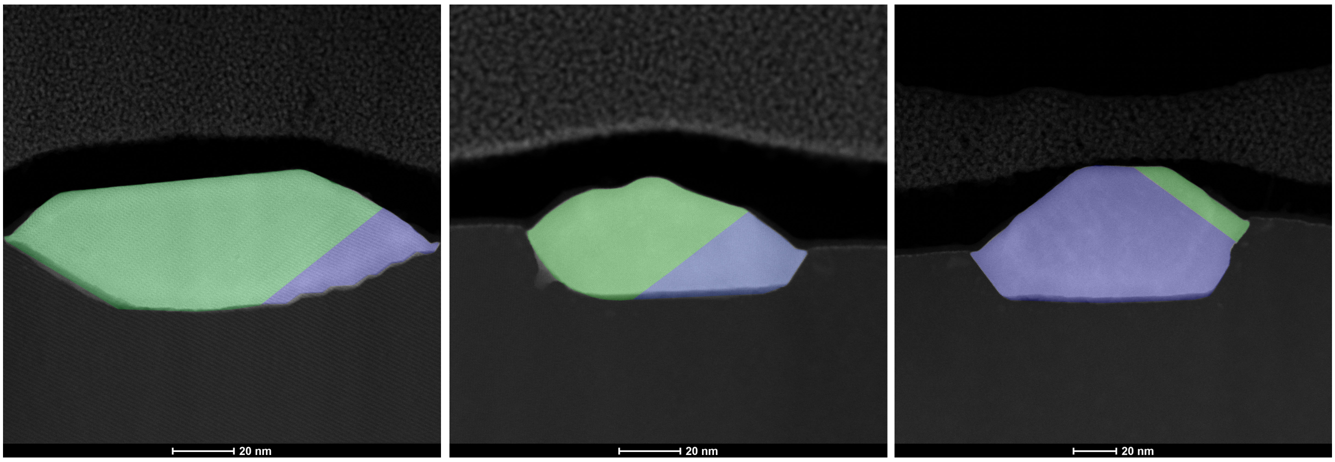

Figure S11: Three representative HAADF-STEM images of FIB sectioned lamellas from the sample annealed at 773 K for three different cooling rates, (a) 700 K/min, (b) 15 K/min (c) 0.1 K/min, the islands are viewed along the [110]Au$_{fcc}$/[100]Au$_{hcp}$ direction. The sample prepared at the fastest cooling rate again shows a large content of Au$_{fcc}$ and Au$_{hcp}$ intergrowth but at 773K the unfaulted Au$_{hcp}$ is not observed. For the slower cooling rates the Au$_{fcc}$ content (blue) is increased at the expense of the faulted domain (green)

References

[1] Huang, X., Li, S., Huang, Y., Wu, S., Zhou, X., Li, S., Gan, C. L., Boey, F., Mirkin, C. A. and Zhang, H. Synthesis of hexagonal close-packed gold nanostructures. *Nature Communications* **2**, 292- (2011).

[2] Marshall, A. F., Thombare, S. V. and McIntyre, P. C. Crystallization Pathway for Metastable Hexagonal Close-Packed Gold in Germanium Nanowire Catalysts. *Crystal Growth & Design* **15**, 3734-3739 (2015).

[3] Okamoto, H. and Massalski, T. The Au-Ge (Gold-Germanium) system. *Bulletin of Alloy Phase Diagrams* **5**, 601-610 (1984).

[4] Blumenstein, C., Schafer, J., Mietke, S., Meyer, S., Dollinger, A., Lochner, M., Cui, X. Y., Patthey, L., Matzdorf, R. and Claessen, R. Atomically controlled quantum chains hosting a Tomonaga-Luttinger liquid. *Nat Phys* **7**, 776-780 (2011).

[5] Krok, F., Kaspers, M. R., Bernhart, A. M., Nikiel, M., Jany, B. R., Indyka, P., Wojtaszek, M., Moeller, R. and Bobisch, C. A. Probing the electronic transport on the reconstructed Au/Ge(001) surface. *Beilstein Journal of Nanotechnology* **5**, 1463-1471 (2014).

[6] Van Aert, S., Verbeeck, J., Erni, R., Bals, S., Luysberg, M., Van Dyck, D. and Van Tendeloo, G. Quantitative atomic resolution mapping using high-angle annular dark field scanning transmission electron microscopy . *Ultramicroscopy* **109**, 1236 - 1244 (2009).

[7] Martinez, G. T., Rosenauer, A., De Backer, A., Verbeeck, J. and Van Aert, S. Quantitative composition determination at the atomic level using model-based high-angle annular dark field scanning transmission electron microscopy . *Ultramicroscopy* **137**, 12 - 19 (2014).
